# Urine Recirculation Improves Hemodynamics and Enhances Function in Normothermic Kidney Perfusion

**DOI:** 10.1097/TXD.0000000000000985

**Published:** 2020-03-13

**Authors:** Annemarie Weissenbacher, Daniel Voyce, Carlo D.L. Ceresa, Maria F. Soares, Ian S. Roberts, James P. Hunter, Andrew Cook, Rutger J. Ploeg, Constantin C. Coussios, Peter J. Friend

**Affiliations:** 1 Oxford Transplant Centre, Nuffield Department of Surgical Sciences, University of Oxford, Oxford, United Kingdom.; 2 Department of Visceral, Transplant and Thoracic Surgery, Medical University of Innsbruck, Innsbruck, Austria.; 3 OrganOx Limited, Oxford Science Park, Oxford, United Kingdom.; 4 Department of Cellular Pathology, Oxford University Hospitals NHS Foundation Trust, John Radcliffe Hospital, Oxford, United Kingdom.; 5 Institute of Biomedical Engineering, University of Oxford, Oxford, United Kingdom.

## Abstract

The study compares urine recirculation (URC) to urine replacement (UR) with Ringer’s lactate in a porcine normothermic kidney machine perfusion (NMP) model using a preclinical prototype device.

**Methods.:**

Kidney pairs were recovered uninjured (as live-donor nephrectomy) and perfused consecutively. Pig kidneys (n = 10) were allocated to either NMP with URC (n = 5) or NMP with volume replacement (n = 5). Cold ischemia time was either 2 or 27 hours for the first or second perfusion (URC or UR) of a kidney pair. An autologous blood-based perfusate, leukocyte-filtered, was used and NMP performed up to 24 hours. Perfusion parameters, biochemistry/metabolic parameters were monitored and samples collected.

**Results.:**

Physiological mean arterial pressures and flows were achieved in both groups but were sustainable only with URC. Significantly higher arterial flow was observed with URC (326.7 ± 1.8 versus 242.5 ± 14.3 mL/min, *P* = 0.001). Perfusate sodium levels were lower with URC, 129.6 ± 0.7 versus 170.3±2.7 mmol/L, *P* < 0.001). Stable physiological pH levels were only observed with URC. Perfusate lactate levels were lower with URC (2.2 ± 0.1 versus 7.2 ± 0.5 mmol/L, *P* < 0.001). Furthermore, the hourly rate of urine output was lower with URC and closer to physiological levels (150 versus 548 mL/h, *P* = 0.008). Normothermic kidney perfusion with URC was associated with longer achievable durations of perfusion: the objective in all experiments was a 24-hour perfusion, but this was not achieved in every case. The mean perfusions were 17.3 ± 9.2 hours with URC versus 5.3 ± 1.3 hours NMP with UR; *P* = 0.02. There appeared to be no differences in baseline tubular condition with and without URC.

**Conclusions.:**

URC facilitates long-term kidney NMP in a porcine model. Perfusate homeostasis and stability of renal arterial flow throughout the perfusion period was only achievable with URC, independent of cold ischemia time duration.

## Abstract 2

Supplemental Digital Content is available in the text.

Normothermic kidney machine perfusion (NMP) provides possibly several potential advantages over both static cold storage and hypothermic machine perfusion. There is evidence from large animal studies that NMP may be valuable in the assessment and preservation/reconditioning of damaged kidneys before transplantation. Although there appears to be significant benefit in the short periods of NMP studied by the Nicholson/Hosgood group,^1-6^ there is also evidence from the Selzner group that longer periods of NMP may be more beneficial.^7-10^ This corroborates the evidence from clinical trials now published in transplant studies of liver NMP.^11,12^ We previously published the first experience perfusing discarded human kidneys for up to 24 hours. For the first time, urine recirculation (URC) was used to control perfusate volume and electrolyte homeostasis: this has the added advantage of facilitating volume replacement in what, ideally, should be developed as a stand-alone transportable perfusion device.^13^

The aim of the currently described study was to investigate NMP on uninjured porcine kidneys, with the added advantage of comparing URC and urine replacement (UR) in pairs of kidneys from the same donors. This would enable a direct comparison between URC and Ringer’s lactate replacement.

## MATERIAL AND METHODS

### Porcine Kidneys for Perfusion

This study was performed at the Oxford University Biomedical Services, University of Oxford in accordance with the United Kingdom Animal Protection Act 1986; home office project license number is PPL30/3303. Kidney procurement was performed on 5 white female landrace pigs with a weight of 40–50 kg. The 10 kidney grafts recovered (open live-donor nephrectomy procedure) underwent normothermic perfusion at the Institute of Biomedical Engineering, University of Oxford, using the preclinical prototype device described previously.^13^

### Perfusion Protocol

Both kidneys from each donor animal were perfused normothermically, with 1 kidney being perfused without URC (UR group) and 1 with URC (URC group). The 2 kidneys from each donor animal were perfused consecutively, with the second kidney being stored on ice until completion of the first perfusion, resulting in an NMP start after either 2 or 27 hours of cold ischemia time (CIT).

The shorter duration (2 h) was based on the time needed from clamping the renal arteries in the donor to starting of the first perfusion (allowing time to transport the organ between laboratories on ice). The second duration of ice storage was to allow to complete 1 perfusion and set up the second (there was only 1 perfusion system available). The allocation of kidneys into groups (UR and URC) alternated to ensure equality between the groups with respect to CIT.

The perfusion circuit was assembled and primed with 500 mL autologous whole blood from the same donor animal as the kidney, treated first with a leukocyte filter (HAEMONETICS RS1). Before connecting the kidney, while waiting for the system to reach 38°C, the perfusate was supplemented with bolus doses of 5000 units of unfractionated heparin, cefuroxime, and calcium gluconate. For glucose control, blood glucose measurements were carried out within 30 minutes after perfusion start and hourly thereafter. A bolus injection of 2.5 mL of a lipid-free parenteral nutrition solution was administered once blood glucose level dropped below 4 mmol/L. To optimize microperfusion and to overcome the initial vasospasm of porcine renal kidneys, a combination of 3 vasodilators was used; epoprostenol sodium, verapamil, and glyceryl trinitrate. In kidney perfusions without URC, the cannulated ureter was allowed to drain into a graduated measuring cylinder, and Ringer’s lactate was infused to replace the excreted urine as a 1:1 volume replenishment in 20 mL intervals. Reasons for termination of the kidney perfusions were any of (1) arterial flow 50 mL/min or less; (2) pH <7 or >7.7 measured at a Pco_2_ level of 5; or (3) sudden cessation of urine production.

Hemodynamic control was based on mean arterial pressure (MAP) and automatically^13^ maintained in the range of 90–100 mm Hg, representing physiological MAP in a pig. Also as previously described, urine production was continuously monitored using an in-line flow sensor and arterial blood gas analysis comprised an in-line blood gas analyzer.^13^ An i-STAT (i-STAT Portable Clinical Analyzer, Abbott Laboratories, Abbott Park, IL) blood analyzer system was used for hourly glucose, sodium, and lactate measurements.

### Perfusate, Urine, and Tissue Samples

Perfusate samples were taken at 3 different time points: (1) after priming of the circuit with blood and using the leukocyte filter before any supplements were added; (2) after 2 hours of normothermic perfusion; and (3) at the end of perfusion.

Perfusates were analyzed for kidney injury molecule 1 (KIM-1) and neutrophil gelatinase-associated lipocalin (NGAL). This was done by performing an ELISA, according to manufacturer’s instructions. An ELISA kit from FineTest (www.fn-test.com) was used with a detection range of 0.156–10 ng/mL and a minimum detectable dose of <0.094 ng/mL KIM-1. For NGAL, an ELISA kit from DLdevelop (www.dldevelop.com) was used with a detection range of 1.56–100 ng/mL and a minimum detectable level of <0.64 ng/mL.

Urine was collected within the first 15–30 minutes after perfusion start and 2-hourly thereafter, as long as there was urine production.

Core needle biopsies were taken before connecting the kidney to the perfusion circuit, after 2 hours of NMP, and at the end of perfusion/after 24 hours of perfusion. Core needle biopsy specimens were fixed in Millonigs solution and processed for paraffin embedding. Three micrometer-thickness sections were stained by hematoxylin and eosin. The extent of acute tubular injury at both the start and the end of perfusion was measured by adapted scoring for human kidneys according to our institutional pathologists. Evaluation was based on following criteria: 0—absent; 1—loss of brush borders/vacuolation of tubular epithelial cells; 2—cell detachment/cellular casts; and 3—coagulative necrosis.

### Statistical Analysis

Time-averaged longitudinal values of measurements over the course of the perfusion are presented as means and SD and displayed in line charts. The normal distribution was assessed with Q-Q plot and the Shapiro-Wilk test. Linear mixed-effects models were fitted according to the design of the experiment including repeated measures of kidney and hours (perfusion duration) clustered by pig (subject). In these models, the effect of URC and CIT was assessed on each outcome measure. For univariate comparisons, unpaired *t*-tests and Mann-Whitney tests were applied. A *P*-value of <0.050 was considered statistically significant. IBM SPSS Statistics Version 23 and GraphPad Prism 7 were used to perform statistical analyses.

## RESULTS

### Hemodynamic and Metabolic Function Parameters

**FIGURE 1.**
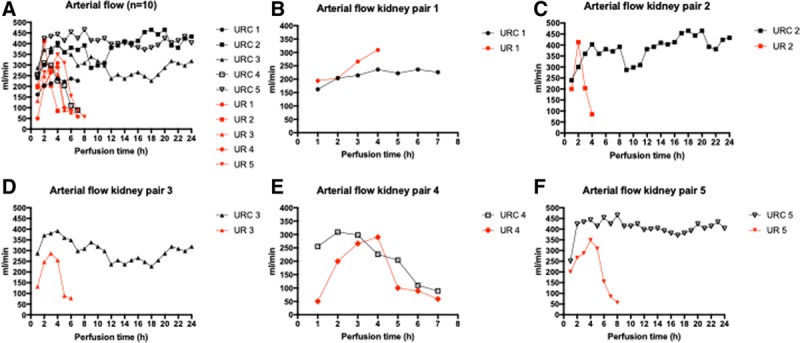
A–F, Arterial flow (n = 10). Arterial flow in mL/min in kidney perfusions with (black) and without (red) urine recirculation. URC and UR with the same numbers are a kidney pair. Kidney pairs are shown in B–F separately. UR, urine replacement; URC, urine recirculation. Cold ischemia times for kidneys were URC 1: 2 h and UR 1: 27 h; UR 2: 2 h and URC 2: 27 h; URC 3: 2 h and UR 3: 27 h; and UR 4: 2 h and URC 4: 27 h; and UR 5: 2 h and URC 5: 27 h.

**FIGURE 2.**
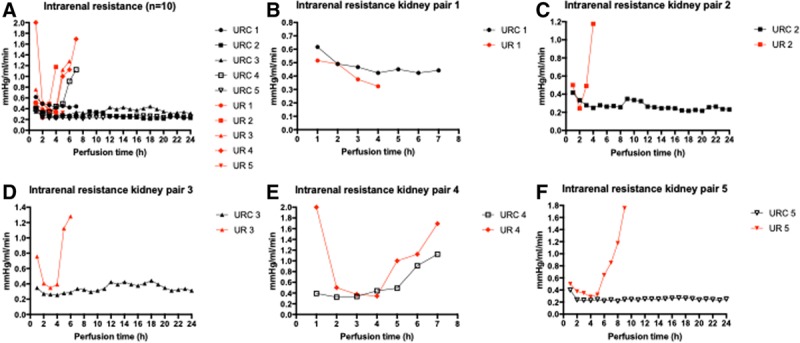
A–F, Intrarenal resistance (n = 10). Intrarenal resistance of kidneys perfused with (black) and without (red) urine recirculation. URC and UR with the same numbers are a kidney pair. Kidney pairs are shown in B–F separately. UR, urine replacement; URC, urine recirculation. Cold ischemia times for kidneys were URC 1: 2 h and UR 1: 27 h; UR 2: 2 h and URC 2: 27 h; URC 3: 2 h and UR 3: 27 h; and UR 4: 2 h and URC 4: 27 h; and UR 5: 2 h and URC 5: 27 h.

Perfusion parameters for all kidneys (n = 10), with and without URC, are displayed in Table 1. Cold ischemia and overall NMP times for each single kidney are displayed in Table 2. Physiological MAPs and flows were achieved in both groups within the first hour of perfusion. These remained stable throughout the whole perfusion period in 4/5 (80%) kidneys perfused with URC. In contrast, sustained physiological parameters were not achieved in any of the kidneys with UR. The hemodynamic parameters are shown graphically in Figure 1A–F (arterial flow) and Figure 2A–F (intrarenal resistance [IRR]).

**TABLE 1.**
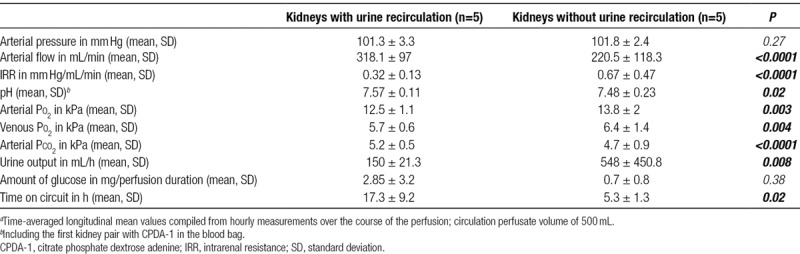
Perfusion characteristics^*a*^

**TABLE 2.**
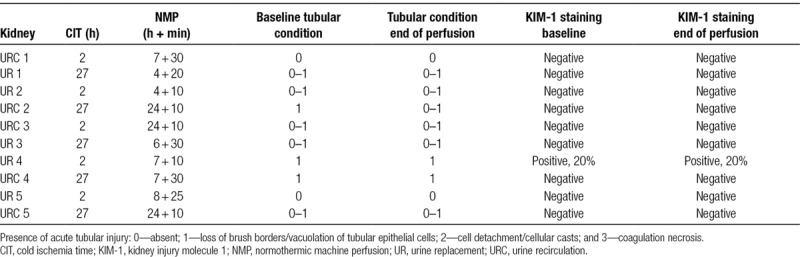
Kidney demographics and histology results

### Arterial Flow

Perfusion failure (defined as perfusate flow of <50 mL/min), occurred in 1 URC kidney after 7 hours. In contrast, perfusion failure occurred in 4/5 (80%) in the group without URC, occurring between 6 and 9 hours of NMP.

The duration of CIT before start of NMP (2 or 27 h) had no negative impact on arterial flow, neither in the overall cohort (n = 10) nor in the groups with or without URC. Therefore, we did not stratify the results solely for short or long CIT or for URC and UR. The linear mixed-effects models (presented in Table 3) assessed the effect of URC and CIT on flow and revealed significant higher arterial flow in the URC group with 326.7 ± 1.8 mL/min compared with the UR group with 242.5 ± 14.3 mL/min; *P* = 0.001. The difference in mean arterial flow between URC versus UR was more pronounced in the kidneys with 27 hours of CIT which had also better arterial flow; *P* for interaction 0.005.

**TABLE 3.**
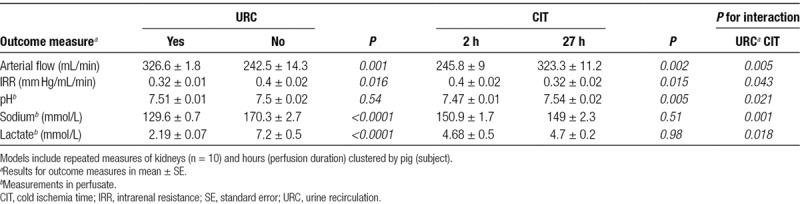
Marginal means of outcome measures estimated by linear mixed-effects models

### Intrarenal Resistance

IRR was expressed in mm Hg/mL/min and calculated by dividing pressure in mm Hg by the flow in mL/min at the same time point. The changes of IRR over time are shown in Figure 2A–F. The average IRR according to the mixed-effects models was significantly lower in the URC group with 0.32 ± 0.01 mm Hg/mL/min compared with 0.4±0.2 mm Hg/mL/min; *P* = 0.016. The difference between IRR in URC and UR was also more pronounced in the kidneys with longer CIT; *P* for interaction 0.043. Data are shown in detail in Table 3.

### pH Levels

**FIGURE 3.**
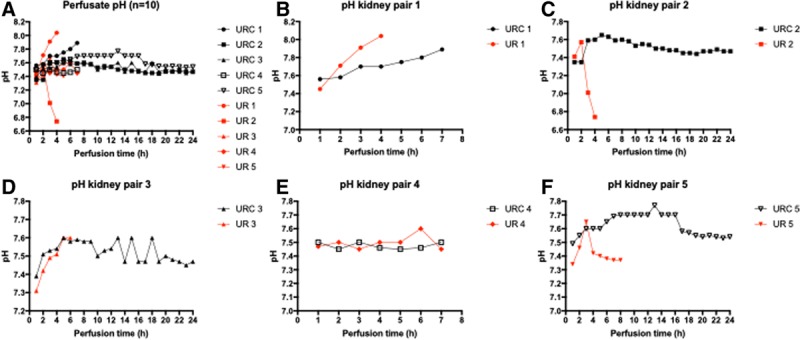
A–F, Perfusate pH (n = 10). pH values of kidneys perfused with (black) and without (red) urine recirculation. URC and UR with the same numbers are a kidney pair. Kidney pairs are shown in B–F separately. Pair 1 had CPDA-1 in the blood collection bag. CPDA-1, citrate phosphate dextrose adenine; UR, urine replacement; URC, urine recirculation. Cold ischemia times for kidneys were URC 1: 2 h and UR 1: 27 h; UR 2: 2 h and URC 2: 27 h; URC 3: 2 h and UR 3: 27 h; and UR 4: 2 h and URC 4: 27 h; and UR 5: 2 h and URC 5: 27 h.

The course of pH for all perfused kidneys is shown in Figure 3A–F. All pH measurements were performed at physiological Pco_2_ pressures of 5 kPa. The perfusate consistently had a starting pH (mean ± SD) of 7.43 ± 0.08 at 38°C, prior to kidney connection. There was no significant difference in the pH of the autologous blood stored for 27 hours, compared with blood stored for 2 hours; 7.39 ± 0.08 versus 7.46 ± 0.07, *P* = 0.22.

Comparison of pH within each individual kidney pair, analyzed by URC/replacement did not show differences. Average pH in kidneys with URC was 7.51 ± 0.1 versus 7.49 ± 0.02 in UR perfusions; *P* = 0.54. Perfusate became more alkalotic in kidneys after CIT of 27 hours; *P* = 0.005. The difference in the mean pH between URC and UR was more pronounced in kidneys with 27 hours of CIT; *P* for interaction 0.021. Data displayed in Table 3.

Notably, in the first kidney pair, a pH > 7.7 was observed at hour 5 in the kidney with URC and 2 hours of CIT, and at hour 4 in the kidney without URC and 27 hours of CIT. Both kidneys started perfusion with a pH > 7.45. We hypothesized that a potential reason for this increasing pH over time was the metabolism of citrate, which is part of the anticoagulant citrate phosphate dextrose adenine (CPDA-1) in the COMPOFLEX blood bag; citrate is used to prevent coagulation by chelating calcium. Each blood bag contains 63 mL CPDA-1 including 1.7 g sodium citrate dihydrate (26.3 g/1000 mL). Each citrate molecule metabolized in a functioning kidney generates 3 molecules of bicarbonate causing metabolic alkalosis of the perfusate.^14-17^ To investigate the hypothesis that the citrate metabolism is the substrate leading to an alkalotic perfusion solution, the 63 mL CPDA-1 was removed from the blood bags and the bags reprimed with 10 000 units of unfractionated heparin and 20 mL of saline. Comparison of the pH levels of the first 2 kidney perfusions with citrate and the subsequent 8 kidney perfusions without CPDA-1 showed a significant difference; pH 7.72 ± 0.16 versus 7.46 ± 0.17, respectively, *P* < 0.0001. Based on this observation, CPDA-1 was removed for all of the remaining kidney perfusions.

### Lactate Levels During Perfusion

When lactate levels are compared (mean ± SD) within each kidney pair, the perfusates with URC consistently showed lower lactate levels.

Listing the URC kidney first in each case, the numerical data are as follows: first pair: 4.35 ± 1.5 versus 5.99 ± 4.12 mmol/L; second pair: 1.13 ± 0.11 versus 11.64 ± 9 mmol/L; third pair: 2.55 ± 1.28 versus 6.9 ± 1.6 mmol/L; fourth pair: 1.87 ± 0.64 versus 9.07 ± 3.98 mmol/L; and fifth pair: 1.39 ± 0.74 versus 6.49 ± 3.2 mmol/L.

According to the mixed-effects models, Table 3, lactate was significantly lower in URC perfusates with 2.19 ± 0.07 mmol/L compared with UR perfusates with 7.17 ± 0.5 mmol/L; *P* < 0.0001. CITs did not interfere with lactate levels. The relationship of URC/UR and CIT length is significant; *P* for interaction 0.018.

### Urine Excretion and Sodium Levels in Perfusate and Urine

All kidneys produced urine. The median hourly urine production of all the kidneys with URC was 143 mL (123–175 mL), compared with 350 mL (293–1350 mL) in kidneys without URC.

**FIGURE 4.**
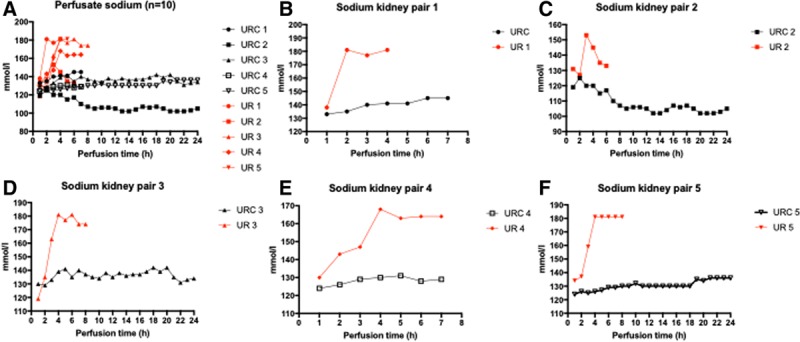
A–F, Perfusate sodium (n = 10). Perfusate sodium levels of kidneys perfused with (black) and without (red) urine recirculation. URC and UR with the same numbers are a kidney pair. Kidney pairs are shown in B–F separately. UR, urine replacement; URC, urine recirculation. Cold ischemia times for kidneys were URC 1: 2 h and UR 1: 27 h; UR 2: 2 h and URC 2: 27 h; URC 3: 2 h and UR 3: 27 h; and UR 4: 2 h and URC 4: 27 h; and UR 5: 2 h and URC 5: 27 h.

The initial sodium levels in the perfusate were higher (164.6 ± 0.6 mmol/L sodium in blood) in the first 2 perfusions (with CPDA-1 anticoagulant) than in all subsequent perfusions (143.6 ± 3.5 mmol/L sodium in blood). There were no differences in the initial sodium levels between the blood cold stored for 2 versus 27 hours (before NMP was initiated). The course of sodium serum levels for each single kidney is displayed in Figure 4A–F.

Perfusate sodium levels of kidneys with URC were lower throughout the perfusion compared with those in kidneys without URC 129.6 ± 0.7 versus 170.3 ± 2.8 mmol/L; *P* < 0.0001. Throughout the perfusion period, perfusate sodium was not significantly influenced by duration of CIT; *P* = 0.51. URC/UR and CIT are significantly linked regarding perfusate sodium; *P* for interaction 0.001.

CIT had an impact on initial urine sodium levels. Urine samples taken within 30 minutes after perfusion start revealed higher sodium levels in the 27 hours CIT group (138.2 ± 9.2 mmol/L), compared with the short CIT group (101.6 ± 8.3 mmol/L; 95% confidence interval, 23.81-49.35 [*P* = 0.008]). As in the perfusate, sodium levels in urine were significantly lower in the group with URC; 94.9 ± 8 versus 117.6 ± 17.6 mmol/L, 95% confidence interval, 10.12-35.15 (*P* < 0.001).

### Urine Protein Levels

Initial urine protein levels from urine samples taken within the first 30 minutes after perfusion start were significantly lower in the short CIT group (1100 ± 243.5 mg/L) compared with the long CIT group (3467 ± 338.3 mg/L), *P* = 0.0006. Urinary protein levels diminished thereafter: in the URC group, the values at 1 hour were 1344 ± 834.9 mg/L and from hour six 362.2±165.1 mg/L. In the group with UR, the 1-hour values were 1528 ± 1435 mg/L and from hour 4 onwards 242.9 ± 126.8 mg/L.

### Histology Results

Table 2 displays the histology results of all perfused kidneys with URC and UR including their demographics accordingly. The baseline tubular condition was described in all time-0 kidney biopsies. The tubular condition before perfusion start was slightly inferior in kidneys with 27 hours of CIT compared to those with 2 hours (scores 0–1), but still scored as only minimal injury, with a score of 1 at the highest. The initial tubular condition appeared unchanged or slightly improved after normothermic perfusion (Table 2).

**FIGURE 5.**
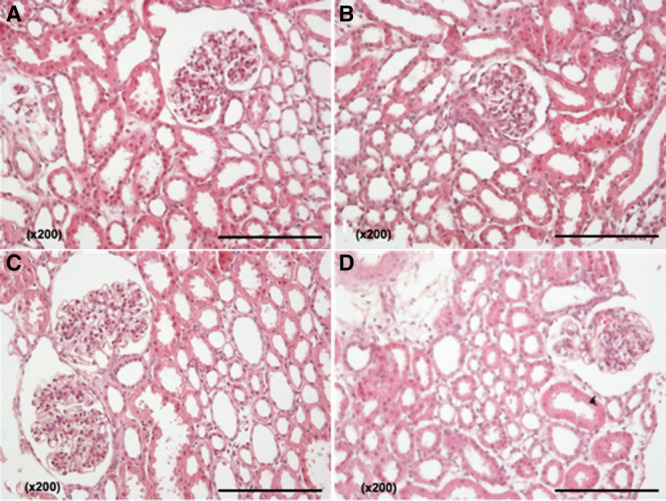
A–D, Photographs of H&E staining (×200 magnification). A, Kidney UR 2, 0 biopsy after 2 h CIT; (B) kidney UR 2, after 4 h of NMP without urine recirculation; (C) kidney URC 2, 0 biopsy after 27 h CIT; and (D) kidney URC 2, after 24 h NMP with urine recirculation. CIT, cold ischemia time; NMP, normothermic machine perfusion; UR, urine replacement; URC, urine recirculation.

Figure 5A–D shows examples of histological findings for kidneys perfused with and without URC.

The fourth pair of kidneys did not perfuse as well as the other pairs, and it was not possible to perfuse either kidney for longer than 8 hours either without or with URC.

Time 0 biopsies of both kidneys showed a severe interstitial nephritis, more marked in kidney UR 4 than in kidney URC 4, with patchy multifocal plasma cell-rich mononuclear infiltrates. In UR 4, the interstitial inflammation intensified during NMP (multifocal plasma cell-rich mononuclear infiltrate, with eosinophils), whereas it showed improvement in kidney URC 4; Figure S1A–D, SDC, http://links.lww.com/TXD/A245.

### KIM-1 Immunohistochemistry

KIM-1 immunoexpression became only positive in kidney UR 4 over time, which is the kidney with the persisting interstitial nephritis; Figure S2A,B, SDC, http://links.lww.com/TXD/A245. KIM-1 positivity could not be detected in any other biopsy-samples at any time (Table 2).

### Biomarker Results in the Perfusate: KIM-1 and NGAL

The KIM-1 start levels (mean ± SD) were comparable between the 2 CIT groups; 109.1 ± 3.8 ng/mL after 2 hours versus 111.9 ± 5.1 ng/mL after 27 hours CIT, *P* = 0.22.

In all but kidney UR 4, KIM-1 levels decreased over time of NMP. Two-hour KIM-1 levels were lower in the group with URC compared to the perfusions without; 65.7 ± 27.7 versus 107.6±5 ng/mL, respectively, *P* = 0.01. The same applies to the difference between start- and 2-hour values; delta 44.5 ± 25.4 ng/mL in the URC group versus delta 3.2 ± 6.4 ng/mL without; *P* = 0.02.

The perfusion-end levels of KIM-1 did not differ significantly between the URC group and the group with UR. The difference between start- and end-perfusion value of KIM-1 was bigger in the URC group, but not significantly; delta 33.8 ± 44.5 with URC compared to delta 18.9 ± 33.6 ng/mL without, *P* = 0.22.

The length of CIT did not impact the 2-hour KIM-1 levels; 88.8 ± 23 ng/mL after 2 hours CIT versus 84.5 ± 36.8 ng/mL after 27 hours CIT, *P* = 0.7. The end-perfusion KIM-1 level was comparable between the short and long CIT group; 65.6 ± 47 ng/mL after 2 versus 103.1 ± 7.8 ng/mL after 27 hours CIT, *P* = 0.15.

NGAL was only detectable in perfusates of kidney pair 4 with the histological diagnosis of interstitial nephritis. In kidney UR 4, the NGAL level 2 hours after perfusion was 62 and 60 ng/mL at the end of perfusion. In kidney URC 4, the NGAL level was 71 ng/mL 2 hours after perfusion and 43 ng/mL at the end of perfusion.

## DISCUSSION

In our previous publication, we presented encouraging evidence to suggest that the recirculation of urine in kidney NMP was beneficial in respect of maintaining stable perfusion and electrolyte homeostasis.^13^ However, this study was performed using perfusions of human kidneys which had been discarded as unsuitable for clinical transplantation: these were damaged to a substantial but variable degree and, therefore, a heterogeneous cohort. The current study attempts to study the effect of urine circulation in a more consistent model.

Building on the work of previous researchers and using leukocyte-depleted blood-based perfusate,^18,19^ the present study demonstrated for the first time that URC makes it possible to extracorporeally preserve undamaged porcine kidneys normothermically for up to 24 hours. A further finding of the study is that the kidneys preserved with URC were comparable whether they had undergone 2 or 27 hours of CIT prior to NMP for up to 24 hours. Remarkably, arterial flow and IRR reached even better, more physiological values, in kidneys cold stored for 27 hours. Perfusate lactate and sodium levels were not negatively impacted either by duration of CIT. This presents the intriguing possibility of overall preservation times in excess of 50 hours; subject to confirmation of safe transplantation of such kidneys in future studies. A limitation of the study is the sample size. The first pair of kidneys developed and experienced severe alkalosis due to the metabolization of citrate in the blood collection bag, as we suspected. The severe alkalosis was the reason for terminating perfusions earlier as planned. However, kidney URC 1 could kept longer on the device as the acid-base balance deteriorated slower. Also, in the fourth pair of kidneys 24 hours could not be reached, neither with UR nor with URC. Pathology results revealed interstitial nephritis in those kidneys and interestingly, NGAL was only elevated in perfusates of URC 4 and UR 4 with decline of NGAL over time only in the URC 4 samples despite the longer CIT of the URC 4 kidney with 27 hours. Besides the unexpected events in kidney pairs 1 and 4, the differences between the URC and the UR group were still clear and we decided, according to The Three Rs and based on the experience we gained with human kidneys before,^15^ not to increase the number of porcine perfusions.

Although the work of the groups of Nicholson and Selzner^1,2,7-10,18,20^ attest to the benefits of shorter durations of NMP with simple urine volume replacement, we believe that this URC may be of value in enabling longer-term perfusions by facilitating electrolyte and acid-base balance. The composition of the perfusate is a fundamental challenge to maintaining a kidney in a functioning state: the kidney excretes the perfusion volume multiple times over a 24-hour perfusion. Whereas accurate volume replacement is relatively straightforward and is easy to envisage as method whereby this could be automated for a clinical device, the same is not true for electrolytes, which are lost in unpredictable proportions. This leads rapidly to electrolyte and acid-base disruption, sufficient to cause cellular dysfunction.

Ringer’s lactate also includes 130 mmol sodium per liter and has a lactate content of 28 mmol/L. One of the most significant findings in our analysis was that the perfusate sodium levels, and the urine sodium levels, were significantly higher in the perfusion group without URC. High perfusate sodium levels are a possible reason for the decline in arterial blood flow in the group without URC, as vasoconstriction is a natural response of a normal kidney to hypernatremia.^21^

Changes of lactate levels during perfusion are complicated to interpret in a metabolically active kidney. The kidney is the second most major lactate-consuming organ after the liver and plays a major role in lactate metabolism. A correlation between lactate levels, glucose administration, and arterial flow was shown (Supplemental Material 1, SDC, http://links.lww.com/TXD/A245); a greater level of glucose administered correlated with a higher lactate level, and the higher the arterial flow the lower the lactate levels. In addition, as described by Bartlett et al,^22^ hourly urine production correlated with final lactate levels.

The most striking finding, however, was the significant reduction of arterial flow beyond perfusion hour 6 in NMP without URC. A similar perfusion-pattern was described in our previous publication in discarded human kidneys.^13^ It remains unclear whether it is the electrolyte imbalance, the hypernatremia, or the change in the oncotic pressure of the perfusate that is instrumental in the decline of arterial blood flow: this is a question that needs further investigation.

Proteinuria in the donor is a recognized characteristic predictive of kidney graft survival,^23,24^ but there is little evidence in the NMP literature regarding urinary protein. Blum et al,^25^ compared hypothermic and normothermic perfusion in porcine DCD kidneys, showing that proteinuria during simulated reperfusion of both cold and warm preserved kidney groups was similar. Hamar et al^8^ described proteinuria of around 1 g per liter throughout perfusion without showing any trend.

In our study, the amount of urinary protein within the first 30 minutes of NMP was significantly lower in the short CIT group compared with prolonged CIT. Notably, this first measurement is not influenced by the dilutive effect of volume replacement in the non-URC group, making this a directly relevant comparison. This effect of CIT is neither a novel finding, nor surprising, but does reinforce the benefit of avoiding cold ischemia, and the desirability of designing the NMP technology to be transportable as suggested in publications previously.^19^

If normothermic perfusion is to be clinically useful in viability assessment, it will be necessary to identify biomarkers that predict posttransplant performance. In this study, we studied NGAL and KIM-1. The former was only detectable in the kidney pair suffering from interstitial nephritis, showing lower levels of NGAL in the kidney perfused with URC over time, although the relevance of this remains unclear. KIM-1 is a well-recognized kidney injury marker that has been studied in kidney transplantation, and also in NMP.^26-28^ There was a correlation between perfusate KIM-1 levels after 2 hours of NMP with the type of circuit (URC or UR): KIM-1 levels were lower in the 5 kidneys with URC. These differences in KIM-1 levels were based on a small number of experiments which calls for caution in the interpretation of nonparametric *t*-tests.

In summary, we have presented data supporting and going beyond what was published previously. Normothermic organ perfusion has been shown by many investigators to be beneficial in organ preservation and reconditioning, but its application in the field of kidney transplantation has lagged behind that of other organs for unknown reasons. Balanced perfusion as well as the opportunity to operate with a fully automated and transportable kidney perfusion device, represent, in our opinion, 3 main advantages of applying URC in kidney NMP. Further technical development is needed to move long-term kidney NMP from the experimental phase to clinical utility. We believe that resolution of the urine problem may prove to be a useful step in this direction and that this will bring improvements in both function and usability.

## ACKNOWLEDGMENTS

Cordial thank you to the team of the Oxford University Biomedical Services, University of Oxford, for their support and collaboration. Special thanks to our statistical advisor Professor Dr Hanno Ulmer, executive director of the Department of Medical Statistics, Informatics and Health Economics at the Medical University of Innsbruck.

## Supplementary Material


