# Supplementary material for: Urine Recirculation Improves Hemodynamics and Enhances Function in Normothermic Kidney Perfusion
Source: Transplant Direct. 2020 Mar 13;6(4):e541. doi: 10.1097/TXD.0000000000000985 (PMC7144999; doi:10.1097/TXD.0000000000000985)
Supplement: Supplementary file 1 [file txd-6-e541-s001.pdf]

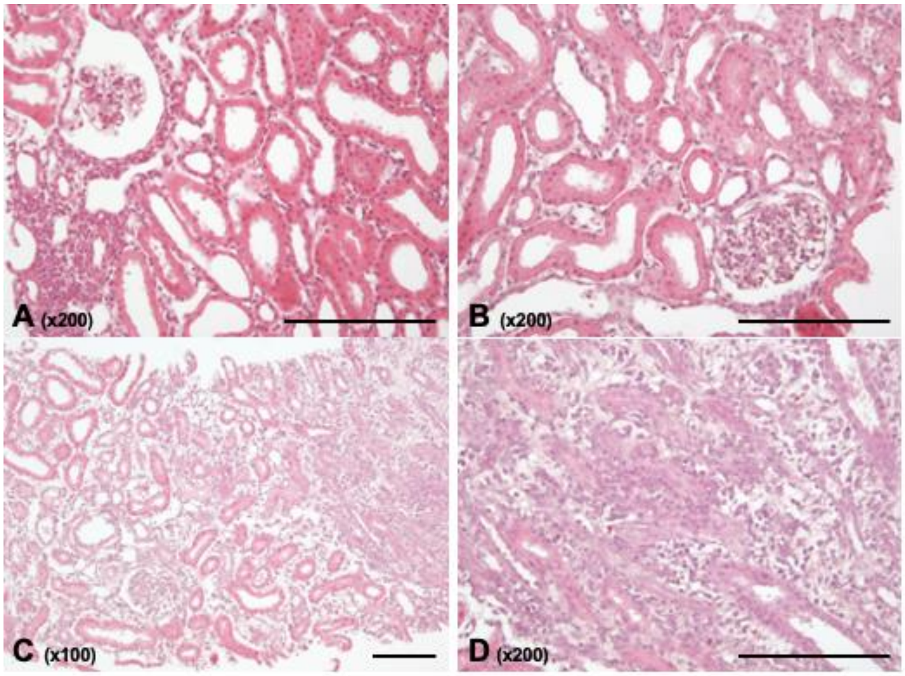

Figure S1A-D: Photograph A shows the 0 biopsy of kidney 'UR 4' with the interstitial inflammation, described as patchy multifocal plasma cell rich mononuclear infiltrate. B illustrates the biopsy after 2 hours of NMP without urine recirculation; C (100x magnification) and D (200x magnification) at the end of perfusion (7 hours) showing the multifocal plasma cell rich mononuclear infiltrate with eosinophils.

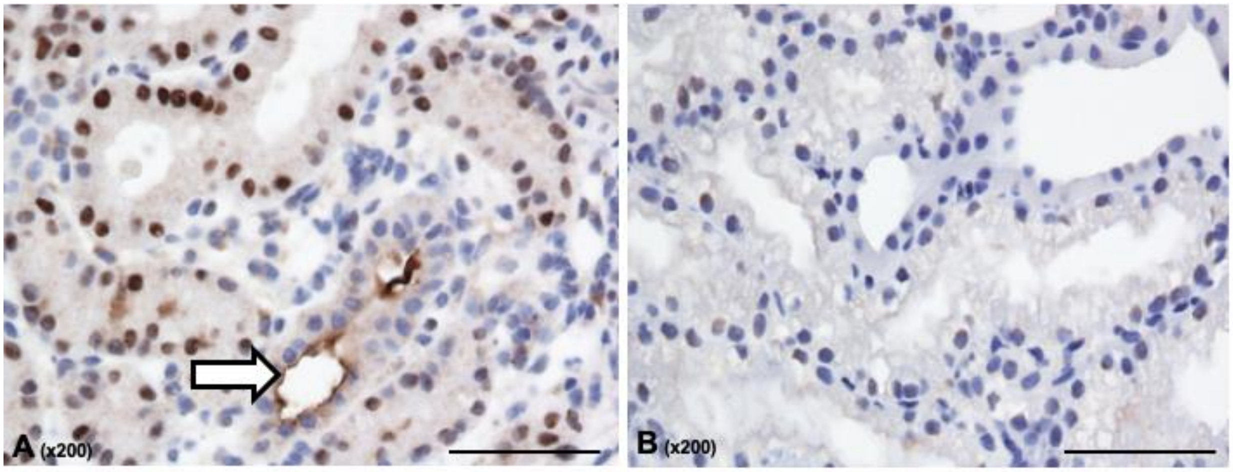

Figure S2A-B: Photograph A shows the KIM-1 positivity (arrow) in kidney ‘UR 4’ at end of perfusion without urine recirculation; photograph B displays KIM-1 negativity in the 24-hour biopsy of kidney ‘URC 2’, with urine recirculation.

Supplemental Material 1: Association of lactate, administered glucose, arterial flow and urine output

In the URC group (n=5), kidneys (1, 3 and 4) with lactate levels higher than the median of 2.51 mmol/l at the end of perfusion received less glucose than kidneys with an end lactate below the median (1.28±1.8 mg vs 3.9±3.76 mg.) Conversely, in the group with urine replacement (n=5), kidneys (2, 3 and 4) with lactate levels higher than the median of 6.99 mmol/l at the end of perfusion received more glucose than kidneys with an end lactate lower than 6.99 mmol/l (1.22±0.94 mg vs 0.33±0.58 mg.)

The median arterial flow throughout the perfusion was lower in kidneys with a lactate level higher than the median of 2.51 mmol/l at the end of perfusion in the URC group; 267±43.8 ml/min vs 339.7±109.3 ml/min. Conversely, in the group with replacement of urine, the flow was higher in kidneys with end-lactate levels higher than the median of 6.99 mmol/l compared to the ones with lower end-lactate levels; 277.5±191.6 ml/min vs 237.3±92.2 ml/min.

The hourly urine volume was lower in kidneys with a lactate level higher than the median of 2.51 mmol/l at the end of perfusion in the URC group; 133±14.1 ml/h vs 161.3±17.9 ml/h. Conversely, in the group with replacement of volume, urine production was greater in kidneys with end-lactate levels higher than the median of 6.99 mmol/l compared to the ones with lower end-lactate levels; 821.5±747.4 ml/h vs 365.7±50.4 ml/h.
